# Impact of COVID-19 on traditional healthcare-associated infection prevention efforts

**DOI:** 10.1017/ice.2020.141

**Published:** 2020-04-16

**Authors:** Michael P. Stevens, Michelle Doll, Rachel Pryor, Emily Godbout, Kaila Cooper, Gonzalo Bearman

**Affiliations:** 1 Hospital Infection Prevention Program, Virginia Commonwealth University Health System, Richmond, Virginia

The coronavirus disease 2019 (COVID-19) pandemic has had an enormous impact on healthcare systems globally. Infection prevention resources at the local level, especially in areas of high SARS-CoV-2 activity, have now been diverted to outbreak management. Although these efforts have understandably taken immediate priority, the impacts on traditional healthcare-associated infection (HAI) surveillance and prevention efforts remain concerning.

A PubMed search utilizing the search terms “Impact of COVID-19 on healthcare associated infections” was performed on April 5, 2020, and yielded no directly applicable results. An informal Twitter poll was initiated on April 4, 2020, asking the infection prevention and hospital epidemiology community what percentage of their traditional infection prevention time had been diverted to COVID-19 response efforts. This query yielded 220 responses: 79.1% indicated spending >75% of their time on COVID-19 response efforts and another 13.2% indicated spending >50%–75% of their time on these efforts. Although these data are limited, they provide a snapshot of the potential resource diversion affecting the infection prevention community, and these percentages are consistent with our local experience.

**Fig. 1.**
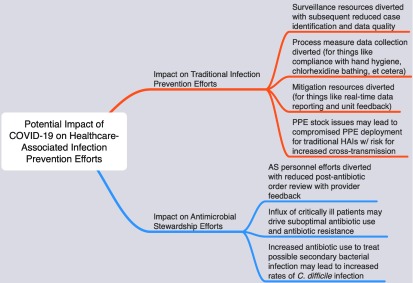
Potential impact of COVID-19 on healthcare-associated infection prevention efforts.

The potential impacts of the diversion of traditional infection prevention resources to focus on the COVID-19 response are numerous (Fig. 1). With regard to more conventional infection prevention duties, surveillance efforts may be compromised leading to compromised case identification. Process measure data collection may also be compromised (eg, compliance with hand hygiene and chlorhexidine bathing, et cetera). Mitigation efforts are also likely to be affected. In the absence of real-time HAI surveillance and provider and unit feedback, an increase in subsequent HAIs is likely.

Another concern is the diversion of traditional personal protective equipment (PPE) resources given significant supply-chain shortages. These shortages resulted in the United States Centers for Disease Control and Prevention (CDC) recommending that hospitals experiencing gown shortages stop using isolation gowns for endemic pathogens such as methicillin-resistant *Staphylococcus aureus* (MRSA), vancomycin-resistant Enterococci (VRE), and extended-spectrum β-lactamase (ESBL)–producing gram-negative rods. The CDC also noted that gown use could be suspended in nonendemic settings for lower-risk patient encounters.^1^ Such strategies may lead, especially if the local healthcare personnel infrastructure is also compromised, to more cross transmission and HAIs.

The impacts on antimicrobial stewardship programs (ASPs), many of which are integrated with infection prevention programs, may also be significant.^2^ ASP efforts may be diverted to assist with COVID-19 response efforts, with a negative subsequent impact on activities such as post-antibiotic order review with provider feedback. An influx of critically ill patients may drive suboptimal antibiotic use with subsequent concern for the development of antibiotic resistance and *C. difficile* infection as well.

Another major concern is the potential impact of increased HAI rates on health systems in terms of public reporting and quality programs associated with reimbursement. Predictably, health systems will be negatively impacted by the global pandemic, and further penalties related to an increase in HAIs will further stress these systems. Although these measures are well-intentioned, consideration for relaxing penalties during the pandemic (and for a period afterward) should be considered. The Centers for Medicare and Medicaid Services (CMS) penalized ~800 hospitals for their HAI performance between October 2018 and September 2019, resulting in a 1% loss of reimbursement for Medicare patients.^3^ Fortunately, the CMS announced that reporting to the hospital-acquired condition (HAC) program is optional for the fourth quarter of 2019. Furthermore, data for the first 2 quarters of 2020 will not be counted for performance or repayment, and the related data do not need to be submitted to the CMS.^4^ Other non-CMS quality programs that utilize publicly reported HAI data to determine penalties should also consider adopting similar nonpenalty measures in the face of the current global health crisis.

Health systems should consider creative ways to support and bolster their infection prevention programs during the COVID-19 pandemic. These efforts should include investment in information technology and personnel. Although health systems may be willing to invest resources and money in these programs, few trained infection preventionists and hospital epidemiologists are available in the United States. Beyond the current pandemic, significant resources should be invested to improve and sustain infection prevention infrastructure at the local, regional and national levels. Additionally, new investment in training and expanding the infection prevention workforce will be critical.

The full impact of the COVID-19 pandemic on health systems and traditional HAIs remains to be determined. Significant infection prevention resource diversion is occurring to help manage the outbreak at the health-system level, which will predictably impact HAI surveillance and prevention efforts. The CMS has recognized the challenges facing the infection prevention community and have suspended penalties associated with the HAC program temporarily. Other quality programs should consider similar measures. Now more than ever, health systems should continue investing in their infection prevention programs (both infrastructure and personnel) beyond the current pandemic.
