# Impact of COVID-19 on Traditional Healthcare Associated Infection Prevention Efforts – CORRIGENDUM

**DOI:** 10.1017/ice.2020.250

**Published:** 2020-10

In the above mentioned published article by Stevens et al^1^, the authors state that the Centers for Medicare and Medicaid Services (CMS) suspended penalties associated with the Hospital-Acquired Condition (HAC) Reduction Program temporarily; it is more accurate to state that the CMS has made data reporting for the fourth quarter of 2019 and the first 2 quarters of 2020 optional. For the HAC Reduction Program, “if data from January 1, 2020 - March 31, 2020 (Q1) is submitted, it will be used for scoring in the program.”^2^
